# Long-term complications in patients with bladder-prostate rhabdomyosarcoma treated with brachytherapy: a systematic review

**DOI:** 10.1007/s00383-025-06112-9

**Published:** 2025-09-09

**Authors:** Giulia Brooks, Filippo Ghidini, Calogero Virgone, Federica De Corti

**Affiliations:** 1 Department of Pediatric Surgery and Pediatric Minimally Invasive Surgery and New Technologies, San Bortolo Hospital, Viale Ferdinando Rodolfi 37, 36100 Vicenza, Italy; 2 Department of Women’s and Children’s Health, University of Padua, Via Nicolò Giustiniani, 35100 Padua, Italy; 3 Department of Pediatric Surgery, Hôpitaux Civils de Colmar, Pasteur 2, 1 Rue Dr Paul Betz, 68000 Colmar, France; 4 Pediatric Surgery Unit, Department of Women’s and Children’s Health, University of Padua, Via Nicolò Giustiniani, 35100 Padua, Italy

**Keywords:** Brachytherapy, Rhabdomyosarcoma, Children, Long-term outcomes

## Abstract

**Introduction:**

Brachytherapy has been used for the multimodal treatment of pediatric bladder-prostate rhabdomyosarcoma in the last two decades. The aim of this systematic review is to gather the current evidence about this innovative technique with a special focus on long-term outcomes.

**Methods:**

According to PRISMA criteria, PubMed, Scopus, and Web of Science were searched for papers published between 2000 and 2022.

**Results:**

The search yielded 7338 papers but only seven were eligible, for a total of 196 children with a median age ranging from 23 to 32 months and a median follow-up ranging from eight to 64 months. The five-year overall survival was superior to 90%. However, at least one complication involving the urogenital apparatus was reported in 66 children (35%).

**Conclusion:**

Brachytherapy presented positive outcomes in terms of overall survival. On the other hand, further efforts should be made to decrease the risk of functional urogenital side effects.

## Introduction

Rhabdomyosarcoma (RMS) is a malignant tumor of mesenchymal origin and is the most common soft-tissue neoplasm in children, accounting for 5% of all pediatric cancers [1]. The overall annual incidence in Europe is 5.4 cases per million (children < 15 years) [2]. The genitourinary apparatus is one of the most frequent localizations, representing 15–20% of cases [3]. More specifically, genitourinary rhabdomyosarcoma commonly involves the bladder and prostate. These organs account for 5% of all pediatric RMS [4]. Peak incidence lies in the first two years of life [5] with a 2:1 male preponderance [6]. Most of the cases belong to the embryonal histotype [7].

In the last decade, the five-year survival reached up to 80%, thanks to multimodal treatment that includes surgery, chemotherapy, and radiotherapy [8]. However, these treatments can be correlated to severe side effects. Castagnetti et al. reported that 60% of patients presented long-term urinary dysfunctions [3]. Therefore, different strategies are adopted to reduce the risk of side effects caused by treatments for the local control of the disease. Firstly, organ-sparing surgery principles have been improved. Secondly, technological innovation aimed to reduce side effects due to radiotherapy by introducing brachytherapy in the treatment protocol [9]. Brachytherapy (from ancient Greek brakhús, short) allows the administration of radioisotopes in a target volume through plastic tubes that are inserted in or near the tumor. This technique allows an effective dose to be administered to the target tissue, reducing healthy tissue damage. Up to now, brachytherapy is available only in highly specialized centers that reported positive short and long-term outcomes [10, 11].

The primary aim of this review was to gather the current evidence about the use of brachytherapy for pediatric bladder-prostate rhabdomyosarcoma (b-p RMS). The secondary aim was to assess the outcomes of this innovative approach for the local control of the disease, with particular emphasis on the different strategies used to limit the potential adverse effects to genitourinary apparatus in both genders.

## Methods

### Protocol

According to the PICO model, the population included all the pediatric patients affected by b-p RMS (*P* population) that underwent brachytherapy regardless of the dose or the irradiation protocol (*I* intervention). This group was compared to the patients affected by the same disease that underwent other multimodal treatment, including surgery, conventional radiotherapy, and proton beam radiotherapy *(C* control*)*, to assess the survival, the risk of relapse, and the long-term functional complications due to the treatment (*O* outcome).

### Search strategy

According to a preliminary search in the PubMed database, no published systematic review was found by the authors about this topic. The systematic review was performed according to an a priori designed protocol which followed the PRISMA (Preferred Reporting Items for Systematic Reviews and Meta-Analyses) [12] reporting guidelines.

PubMed, Scopus, and Web of Science were consulted in March 2023. The following combinations of relevant medical subject heading (MeSH) terms, keywords, and word variants for “pediatric bladder rhabdomyosarcoma”, “pediatric prostate rhabdomyosarcoma”, “pediatric vesicoprostatic rhabdomyosarcoma” and “pediatric rhabdomyosarcoma brachytherapy” were chosen for the search. Reference lists of relevant articles and reviews were further screened for additional papers.

The results of the search were restricted to the period ranging from January 2000 to December 2022. Only papers written in the English language were considered.

### Inclusion criteria and data extraction

Two Authors (GB, FG) independently screened all the abstracts provided by the research results. Agreement about potential relevance was reached by consensus. Full-text manuscripts of the relevant papers were obtained.

We considered only articles dealing with patients with age inferior to 18 years, affected by b-p RMS, and treated with brachytherapy. Case reports, case series with less than five patients, and literature reviews were excluded.

The selection process was reported using the PRISMA flowchart.

Studies were assessed according to demographic variables, site and stage of the tumor, histology, surgical approach and outcomes, gonadal transposition, parameters and outcomes of brachytherapy, overall survival, and relapse.

The raw data were reported in a database created using MS Excel^®^.

### Quality assessment

Two Authors (FG, GB) independently assessed the methodological quality of the included studies according to the Quality Assessment Tool for Case Series Studies [13].

The risk of bias assessment was performed by using the ROBINS-I V2 tools [14].

## Results

**Fig. 1:**
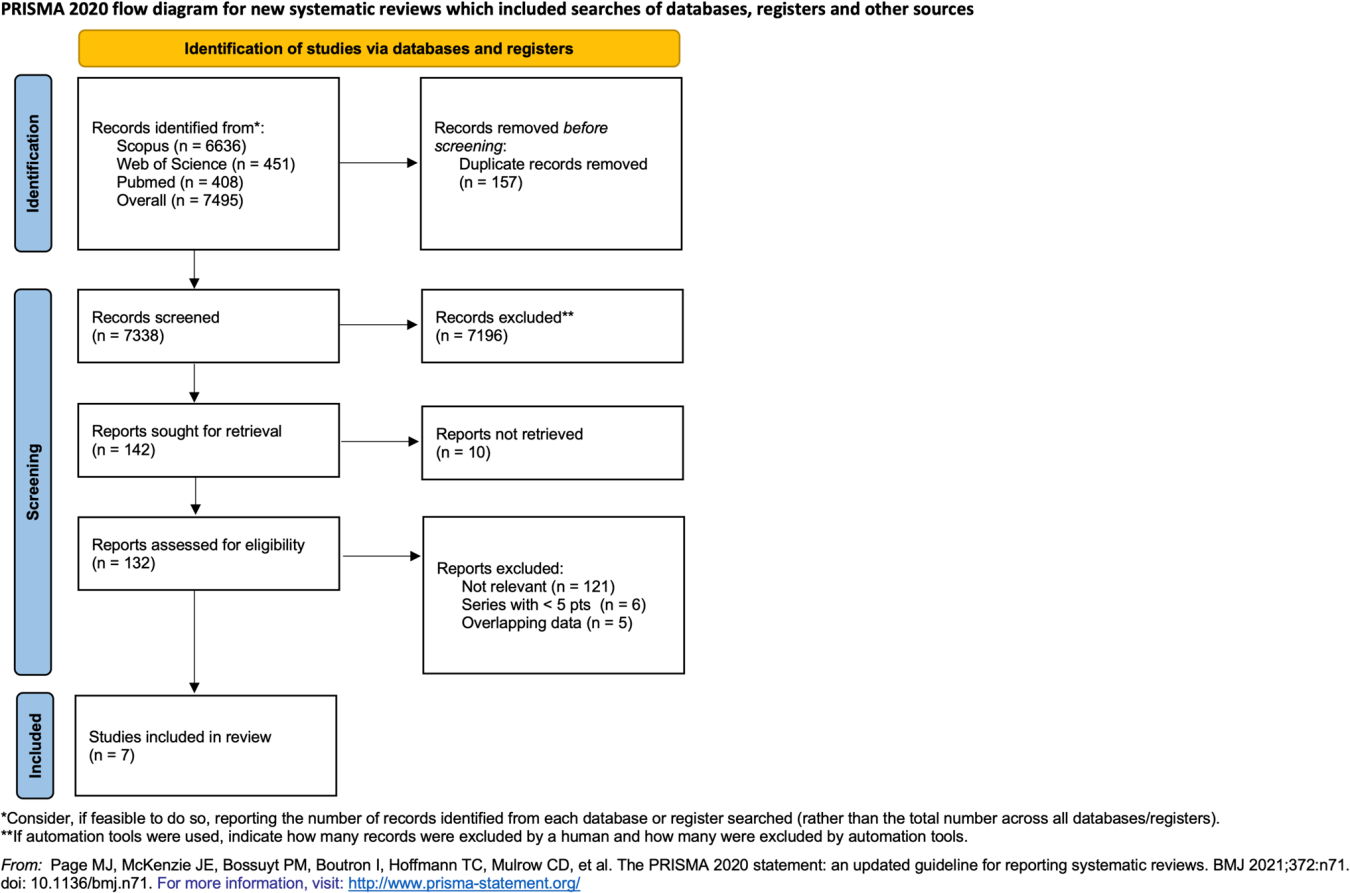
PRISMA flow diagram of the search and screening process

The search strategies yielded 7338 papers. After the selection process displayed in Figure 1, only seven papers [11, 15–20] were eligible for the purpose of the review, and only two of them were prospective. The quality assessment of the included studies is displayed in Table 1. The risk of bias assessment is displayed in Table 2.

**Table 1:** Quality assessment of the included studies according to the NIH quality assessment tool for case series studies

Criteria	Studies						
	Chargari [15]	De Lambert [16]	Schmidt [11]	Limkin [17]	Ellerkamp [18]	Stenman [19]	Lobo [20]
1 Was the study question or objective clearly stated?	Y	Y	Y	Y	Y	Y	Y
2 Was the study population clearly and fully described, including a case definition?	Y	N	N	N	Y	Y	Y
3 Were the cases consecutive?	Y	Y	Y	N	Y	Y	Y
4 Were the subjects comparable?	Y	N	Y	N	Y	N	Y
5 Was the intervention clearly described?	Y	Y	Y	Y	Y	Y	Y
6 Were the outcome measures clearly defined, valid, reliable, and implemented consistently across all study participants?	Y	Y	Y	Y	Y	N	Y
7 Was the length of follow-up adequate?	Y	Y	Y	Y	Y	Y	Y
8 Were the statistical methods well-described?	Y	N	Y	Y	Y	NA	Y
9 Were the results well-described?	Y	Y	Y	Y	Y	N	Y
Overall quality rating	Good	Fair	Good	Fair	Good	Fair	Good

*N* no, *NA* not applicable, *Y* yes

**Table 2:**
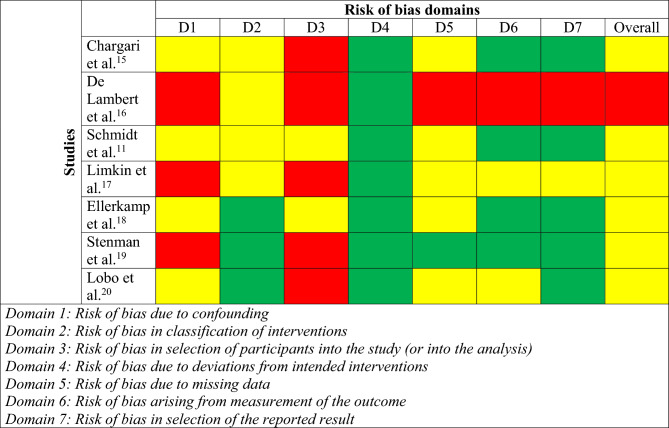
Visualization of the risk of bias assessment

The studies included 196 patients with a median age ranging from 23 to 32 months. Most of them were male. The bladder was the most common tumor site. Most tumors presented an embryonal histology. Other characteristics were summarized in Table 3.

**Table 3:** Clinical and demographic characteristics of the patients of the included studies

Author	Country	Year (study period)	Study design	Sample size (gender)	Age at surgery (median)	Tumor site	Histology	IRS group stage	Tumor size
Chargari et al. [15]	France	2017 (1991-2015)	Prospective	100 (88 M, 12 F)	28 months	36 B 30 P 20 B+P 14 B+U	97 E 3 A	84 III 12 IV 4 relapse	Not available
De Lambert et al. [16]	France	2018 (2016-2017)	Retrospective	8 (8 M, 0 F)	24 months	8 B+P	8 E	8 III	Not available
Schmidt et al. [11]	Germany	2020 (2009-2019)	Retrospective	23 (not available)	26 months	16 B+P 7 B	not available	Not available (1 relapse)	11 < 5 cm 12 > 5 cm
Limkin et al. [17]	France	2021 (2015-2020)	Retrospective	12 (0 M, 12 F)	32 months	12 B	12 E	Not available	Not available
Ellerkamp et al. [18]	Germany Austria	2022 (2009-2022)	Retrospective	30 (29 M, 1 F)	25,5 months	18 B+P 9 B 3 P	20 E 9 A 1 anaplasia	Not available	16 < 5 cm 14 > 5 cm
Stenman et al. [19]	Sweden	2022 (2004-2018)	Retrospective	10 (7 M, 3 F)	27,6 months	4 B+P 3 B 3 P	10 E	10 III	Not available
Lobo et al. [20]	UK	2022 (2014-2019)	Prospective	13 (10 M, 3 F)	23 months	not available	11 E 2 B	Not available	4.3 cm (median)

*M* male, *F* female, *B* bladder, *P* prostate, *B+P* bladder and prostate, *B+U* bladder and urethra, *E* embryonal, *A* alveolar

The data about the initial multidisciplinary treatment before brachytherapy were incomplete in most of the studies. However, it is relevant to report that only a few patients underwent conventional external radiotherapy before brachytherapy. The multidisciplinary approach was described in Table 4.

**Table 4:** Initial multidisciplinary treatment for the patients in the included studies

Author	Chemotherapic protocols	Median number of course of chemotherapy	Initial surgery	Radiotherapy
Chargari et al. [15]	60 IVA +/− doxorubicin 22 IVA +/− carboplatin/topotecan 18 other	6	Not available	5
De Lambert et al. [16]	6 IVA 1 IVA + doxorubicin/carboplatin/topotecan	7,5	Not available	0
Schmidt et al. [11]	Not available	not available	Not available	Not available
Limkin et al. [17]	Not available	6	Not available	0
Ellerkamp et al. [18]	Not available	not available	Not available	Not available
Stenman et al. [19]	10 IVA +/− doxorubicin	6	Not available	5
Lobo et al. [20]	13 IVA	3	13 biopsy	0

*IVA* ifosfamide, vincristine, actinomycin

Eighty patients (41%), belonging to a single series, underwent a low-dose radiation (LDR) protocol, while 76 (39%) underwent a high-dose radiation (HDR) protocol, and the remaining 40 (20%) a pulsed-dose radiation (PDR) protocol. The surgical approach, including the ureteral derivation and the gonadal transposition, was extremely heterogeneous among the included studies. The parameters of the brachytherapy are reported in Table 5.

**Table 5:** Characteristics and parameters of the brachytherapy and associated procedures for the included studies

Author	Brachytherapy	Dose	Tubes/loops	Sessions	Associated surgical procedures	Macroscopic residual tumor (R2)	Ureteral derivation	Gonadal transposition
Chargari et al. [15]	80 LDR 20 PDR	60 Gy (median)	80 2 tubes/loops 20 4 tubes/loops	Not available	38 partial cystectomy 36 partial prostatectomy 13 bladder mucosectomy 7 partial prostactomy + bladder mucoectomy 6 biopsy	63	55 bilateral reimplantation 8 unilateral reimplantation	Not available
De Lambert et al. [16]	8 PDR	60 Gy (median)	Not available	Not available	not available	not available	Not available	8 unilateral testicular transpostion (7 right, 1 left)
Schmidt et al. [11]	23 HDR	30-36 Gy	Not available	Not available	11 not available 9 partial cystectomy 2 biopsy 1 bladder mucosectomy	1	7 bilateral reimplantation 1 transverse ureteroureterostomy	0
Limkin et al. [17]	12 PDR	60 Gy	11 4 tubes/loops 1 5 tubes/loops	Not available	not available	not available	Not available	12
Ellerkamp et al. [18]	30 HDR	36 Gy	Not available	12	10 partial cystectomy 9 partial prostatectomy 1 radical prostatectomy 2 biopsy	2	5 unilateral reimplantation 1 ureteroureterostomy	0
Stenman et al. [19]	10 HDR	39–42 Gy	2–5 tubes/loops	6–16	4 bladder mucosectomy 5 biopsy 1 partial cystectomy	1	2 bilateral reimplantation	3 bilateral ovarian trasposition
Lobo et al. [20]	13 HDR	27,5 Gy	6–18 tubes/loops	5	6 no surgical procedure 4 percutaneous endoscopic poypectomy 3 partial cystectomy	3	0	1 bilateral ovarian trasposition

*HDR* high dose ratem, *LDR* low dose rate, *PDR* pulsed dose rate

The included studies showed a five-year overall survival superior to 90%. Despite this considerable result, 66 patients (35%) presented at least one adverse event affecting the urogenital apparatus. The outcomes and complications of brachytherapy are described in Table 6.

**Table 6:** Outcomes of the patients treated with brachytherapy in the included studies

Author	Median follow-up	Post-operative complications	Adverse events related to brachytherapy	5-year overall survival	5-year disease/event-free survival	Relapse after brachytherapy
Chargari et al. [15]	64 months	2 tube misplacement	14 urinary disfunction 3 kidney injury 3 rectal injury 1 erectile disfunction	91%	84%	6 local relapse 5 local and metastatic relapse 1 metastatic relapse
De Lambert et al. [16]	10 months	1 SSI	not available (no sexual dysfunction reported)	Not available	Not available	Not available
Schmidt et al. [11]	8 months	Not available	9 urinary disfunction (1 long-term)	100%	85,6%	3 local relapse
Limkin et al. [17]	45 months	0 complication	3 proctitis 1 vaginal stenosis	100%	Not available	0 relapse
Ellerkamp et al. [18]	32,5 months	8 urinary leakage 3 actinic urethritis 1 ureteral fibrosis 1 rectourethral fistula	17 urinary disfunction 1 renal injury 1 erectile disfunction	94.7%	74.4%	1 relapse
Stenman et al. [19]	62 months	2 tube misplacement 1 unilateral hydronephrosis 1 rectal perforation 1 urosepsis	3 urinary disfunction 2 delayed puberty 1 renal injury	100%	90%	1 relapse
Lobo et al. [20]	42 months	0 complication	7 urinary disfunction (long-term)	100%	Not available	0 relapse

*SSI* surgical site infection

## Discussion

### Main findings

The treatment of pediatric b-p RMS has greatly changed in the last forty years [21], going from anterior exenteration as the first-line treatment in the 1970 s to a multimodal approach including organ-sparing surgery, chemotherapy, and radiotherapy.

The aim of our study is to report the current evidence concerning the effectiveness and safety of brachytherapy for this subset of patients. The analyzed sample includes 196 patients and, although small, reflects the epidemiological characteristics of b-p RMS. Patients were enrolled in the brachytherapy treatment protocols following selection criteria that varied between centers [10, 11]. The common criterion was a tumor extension that allowed an effective organ-sparing surgery without compromising bladder function. Chemotherapy regimens followed shared international protocols. Few patients underwent external beam therapy before brachytherapy. All surgical approaches are aimed at the preservation of the native bladder and minimizing the irradiation of healthy tissue. Most studies reported the use of ureteral reimplantation, whether prophylactic or reconstructive, while gonadal transposition was performed in a few centers. Brachytherapy vectors were always positioned during surgery. The therapeutic regimen varied between studies: LDR administered at 10 Gy (Gray) daily through manually loaded iridium wires [22], PDR delivered at 1–2 Gy every 1–4 hours avoiding constant irradiation but limiting patients’ movements [14], HDR, which in pediatric patients consists of no more than 7 Gy administered at 8-hour intervals in small volumes [23].

Raney et al. [24] reported the follow-up data concerning 164 patients treated from 1979 to 1998 with multimodal protocols including external beam radiotherapy: adverse long-term effects included urinary incontinence (31% of patients with native bladder and 27% of patients who underwent partial cystectomy), abnormal renal function, gastrointestinal complications, and erectile dysfunction. These complications have a multifactorial etiology, as they can be attributed to the effects of surgical resection, chemotherapy toxicity, or actinic damage.

In this review, 35% of patients presented adverse effects involving the urogenital apparatus, such as urinary incontinence, bladder hyperactivity, vaginal stenosis, and erectile dysfunction.

Lower urinary tract symptoms (LUTS) should be investigated in order to understand their real etiology, their severity, and their potential transitoriness. This is crucial to establish a correct bladder voiding management and follow-up to prevent further complications, such as urinary tract infections and deterioration of kidney functions.

Rectal damage (i.e., rectal ulcer and stenosis) was reported in a few patients.

The data collected by the systematic review were not sufficient to assess the impact of ureteral reimplantation and gonadal transposition on the potential sequelae for the genitourinary system.

The ureteral reimplantation was advocated by most of the authors, in case of the involvement of the trigone, aiming to preserve the anti-reflux mechanism and to avoid actinic injuries that might lead to a fibrotic stenosis [3]. For the same reason, gonadal transposition was introduced to preserve functional parenchyma, even though the procedure was not supported by strong evidence in this peculiar population [20].

While the reported long-term complications are shared with conventional radiotherapy, to our knowledge there are no reported cases of bone growth alterations nor of secondary neoplasms associated with brachytherapy in pediatric b-p RMS.

While our data does not show an increase in long-term outcomes related to brachytherapy, reports regarding 5-year overall survival and relapse rate seem promising. However, more studies are needed to obtain long-term results and compare these patients’ outcomes with patients treated with conventional radiotherapy.

### Limitations

Limitations and bias derive from the design of the studies included in this review. Most studies are retrospective, and the outcome reports are heterogeneous. Moreover, a lack of a control group made any statistical inference impossible. Most of the patients included in the control groups presented with less severe diseases, exposing them to a selection bias. Therefore, a meta-analysis could not be performed, and only a qualitative synthesis was made. Finally, the heterogeneity of brachytherapy protocols among the tertiary centers, together with a lack of comparison between the different multimodal approaches, increased the risk of bias and made the interpretation of the results more difficult. For these reasons, definite conclusions supported by statistical evidence could not be drawn.

### Implications for clinical practice and research

Even though the first goal of the brachytherapy in the multimodal treatment is to avoid a radical cystectomy, a major surgical intervention was associated with the placement of the tubes. Most of the surgeons performed an open approach to perform a partial cystectomy to reduce the tumoral mass and to verify the correct position of the tubes [11, 15–19]. Nevertheless, this surgery might expose one to long-term functional complications, such as a reduced capacity. In only four cases, the tubes were placed through a percutaneous approach associated with an endoscopic polypectomy [20]. This aspect might represent an additional element to be implemented to further reduce the risk of complications associated with major surgery.

Brachytherapy is associated with promising long-term outcomes in terms of survival while reducing the need of mutilating surgery and the irradiation of healthy tissues. The encouraging reports in literature should prompt research groups to develop standardized treatment protocols that include brachytherapy, giving indications regarding its timing, patient inclusion, and regimen of choice. Further efforts must be directed at reducing urogenital long-term side effects, especially by standardizing the indications for ureteral reimplantation, gonadal transposition, and bladder voiding management.

Another interesting implication for future research could be a comparison to the outcomes provided by the proton beam therapy, which is another approach to avoid the consequences of conventional photon radiotherapy. This technique allows concentrating the energy into a preestablished area avoiding diffusion to other deeper tissues. In a recent study, the results were promising with a 5-year local control of 100% for tumors smaller than 5 cm [25].

## Data Availability

The data that support the findings of this study are available on request from the corresponding author, [FG].

## Abbreviations

RMS: Rhabdomyosarcoma
b-p RMS: Bladder-prostate rhabdomyosarcoma
LDR: Low-dose radiation
HDR: High-dose radiation
PDR: Pulsed-dose radiation
LUTS: Lower urinary tract symptoms
